# Curing Effect on Durability of Cement Mortar with GGBS: Experimental and Numerical Study

**DOI:** 10.3390/ma15134394

**Published:** 2022-06-21

**Authors:** Rabih Ghostine, Nicolas Bur, Françoise Feugeas, Ibrahim Hoteit

**Affiliations:** 1 Department of Mathematics, Kuwait College of Science and Technology, Doha 35004, Kuwait; 2 Mechanics Laboratory, University of Lille, 59000 Lille, France; nicolas.bur@univ-lille.fr; 3 ICube, UMR CNRS 7357, INSA Strasbourg, 24 Boulevard de la Victoire, University of Strasbourg, 67084 Strasbourg, France; francoise.feugeas@insa-strasbourg.fr; 4 Applied Mathematics and Computational Science, King Abdullah University of Science and Technology, Thuwal 23955, Saudi Arabia; ibrahim.hoteit@kaust.edu.sa

**Keywords:** durability, permeability, GGBS, curing conditions, Markov chain Monte Carlo

## Abstract

In this paper, supplementary cementitious materials are used as a substitute for cement to decrease carbon dioxide emissions. A by-product of the iron manufacturing industry, ground granulated blast-furnace slag (GGBS), known to improve some performance characteristics of concrete, is used as an effective cement replacement to manufacture mortar samples. Here, the influence of curing conditions on the durability of samples including various amounts of GGBS is investigated experimentally and numerically. Twelve high-strength Portland cement CEM I 52.5 N samples were prepared, in which 0%, 45%, 60%, and 80% of cement were substituted by GGBS. In addition, three curing conditions (standard, dry, and cold curing) were applied to the samples. Durability aspects were studied through porosity, permeability, and water absorption. Experimental results indicate that samples cured in standard conditions gave the best performance in comparison to other curing conditions. Furthermore, samples incorporating 45% of GGBS have superior durability properties. Permeability and water absorption were improved by 17% and 18%, respectively, compared to the reference sample. Thereafter, data from capillary suction experiments were used to numerically determine the hydraulic properties based on a Bayesian inversion approach, namely the Markov Chain Monte Carlo method. Finally, the developed numerical model accurately estimates the hydraulic characteristics of mortar samples and greatly matches the measured water inflow over time through the samples.

## 1. Introduction

Since the end of the tenth century, controlling greenhouse gas emissions has become a major global issue. The production of the ordinary Portland cement (OPC) requires significant energy and releases an important amount of CO${}_{2}$ into the atmosphere. Its manufacturing process generates approximately one ton of CO${}_{2}$ to produce one ton of OPC [1]. It is estimated at around 8% of global CO${}_{2}$ emissions [2,3]. Therefore, cement producers have developed strategies to substitute cement in concrete with other binders to reduce CO${}_{2}$ emissions. The main considered criteria are environmental protection, user’s safety and health, aesthetic, and functionality aspects. Generally, the manufacture of concrete can be optimized to: (1) minimize the consumption of raw materials; (2) enhance durability, deconstruction, and recycling; and (3) limit the impact on the environment.

The use of cementitious materials such as ground granulated blast-furnace slag (GGBS) replacing cement has many environmental and economic advantages such as the reduction of cement consumption, therefore the CO${}_{2}$ emissions needed for its production [4], the valorization of slag that is currently treated as waste, as well as saving of primary energy to achieve the manufacture of eco-friendly cement [5]. Furthermore, the incorporation of GGBS in concrete production offers many mechanical and technical advantages as it enhances the workability [6,7], durability [8,9,10,11], and mechanical properties [12,13] of concrete. In addition, GGBS minimizes the risk of damage caused by alkali-silica reaction and provides better resistance to chloride penetration and sulfate attacks. GGBS is an industrial waste by-product from blast furnaces used to make iron. The main components of GGBS are amorphous calcium, silica, and alumina, which make it suitable to be utilized as a binder in concrete production. However, the introduction of GGBS in concrete leads to changes in their chemical and mineralogical compositions, and also to micro-structural modifications (including porosity and permeability). Therefore, a deeper knowledge of GGBS concrete sustainability is needed.

Over the past few decades, considerable research has been performed to comprehend the performance of concrete incorporating GGBS. Refs. [14,15,16,17] observed that the compressive strength of concrete was improved significantly with the increase of GGBS content up to 40–60%. However, Refs. [18,19,20] showed that concrete incorporating GGBS has less strength than the 100% cement concrete. Some researchers investigated the impact of GGBS on the drying shrinkage behavior of concrete. However, researchers have contradictory results. Refs. [21,22,23] found that incorporating GGBS into concrete exhibits higher drying shrinkage. Nevertheless, Refs. [24,25,26] observed that concrete with 30% GGBS exhibits 20% lower drying shrinkage. Refs. [27,28] showed that the elastic modulus of concrete increases with an increase of GGBS content up to 50%. They also found a strong correlation between the tensile strength of concrete containing GGBS and its compressive strength. Refs. [29,30,31,32,33] concluded that concrete samples containing GGBS exhibit a lower water absorption compared to the ordinary concrete. Refs. [34,35,36,37] revealed that concrete made with GGBS can provide an excellent sulfate attack resistance. Refs. [38,39,40] observed a reduction in chloride penetration with an increase in the replacement level of GGBS. Refs. [7,32] indicated that the acid attack resistance of concrete was improved with the addition of GGBS. Improvements in the tensile strength of concrete containing GGBS were also reported. Refs. [41,42] found that the inclusion of GGBS in concrete achieved higher flexural strength compared to the conventional concrete. However, because of the relatively low permeability of concrete, experimental methods may last weeks or longer to accurately determine the permeability. It is then crucial to accurately infer the hydraulic properties and predict the water absorption through concrete samples using numerical simulation.

Inverse modeling is a mathematical approach for estimating the variables and parameters driving the evolution of a dynamical system. This is usually done by taking measurements of various observable quantities of the system and using physical models to relate these observations to the dynamic variables. Different inverse techniques have been proposed. These can be usually classified between deterministic least-squares optimization methods [43] and Bayesian inference methods [44]. While the deterministic methods seek the parameters that bring the model prediction closer to the data, the Bayesian inversion approach computes the probability distribution functions of the parameters conditioned on available observations, called *the posterior*. This posterior distribution is evaluated as the product of the likelihood of predicting the data and a given prior distribution reflecting our knowledge of the parameters. Given some experimental data and a range of realistic estimations for each parameter, Markov chain Monte Carlo (MCMC) algorithms [45] enable to sample of the posterior distribution of the parameters, which allows computing the best estimate, based on some criteria, and the associated uncertainties. To date, very few studies attempted to use parameters estimation and inverse modeling to characterize the hydraulic properties of concrete [46,47]. Ref. [46] used a deterministic least-squares identification method, while [47] estimated the hydraulic parameters of concrete and mortar using a genetic algorithm. In this paper, the proposed inverse modeling framework is based on Bayesian inference via Markov chain Monte Carlo.

The work reported here sets out to determine experimentally and numerically the impact of curing conditions and GGBS contents on the durability of high-strength Portland cement CEM I 52.5 N. The effect of curing conditions and GGBS proportions on porosity, permeability, and water absorption by capillarity is investigated. To this end, capillary suction experiments were conducted on mortar samples. The evolution of absorbed water by capillary suction is monitored at different times. Thereafter, these observations were used in an inverse modeling process. Finally, a hydraulic model that simulates the water penetration in mortar samples was implemented. The hydraulic properties of the samples are then efficiently estimated by inferring the observations using the MCMC method.

## 2. Materials and Method

### 2.1. Samples

Cements used in this study are CEM I 52.5 N and mixed CEM I 52.5 N—GGBS. Grinding fineness values for CEM I 52.5 N and GGBS are 4700 and 4100 cm${}^{2}$g${}^{-1}$, respectively. CEM I 52.5 N exhibited a mean particle size of 20 μm and GGBS exhibited a mean size of 22.5 μm. Four proportions of GGBS, namely 0%, 45%, 60%, and 80%, are used to make samples. This allows us to explore changes in the microstructures of the samples. The chemical properties of cement and GGBS are listed in Table 1.

A method based on the NF EN 206-1 standard [48] was used to manufacture mortar samples. A similar water/cement ratio of 0.45 was used to secure similar transfer properties, reflected in the porosity, gas permeability, and pore size distribution. Mix proportions of the samples are given in Table 2. Cylindrical samples of 18 mm diameter and 40 mm long were carried out for the capillary absorption tests.

### 2.2. Curing and Drying

After molding, samples were subjected to standard curing conditions (NF EN 196-1) [48] and kept for 24 h. Later, they were taken out from the molds and subjected to a 28 days curing. Three curing conditions were used:

- Standard curing: samples were at $20\pm 1{\;}^{\circ }$C and a relative humidity (R.H.) ≥ 90%.
- Dry curing: samples were at $19\pm 2{\;}^{\circ }$C and R.H. < 50%.
- Cold curing: samples were at $5\pm 2{\;}^{\circ }$C and R.H. ≥ 90%.

Once the samples are obtained, they were dried under vacuum at a pressure less than 13.32 Pa, which is about one hundred times less than the saturation vapor pressure (∼2000 Pa). The pressure gradient between the core sample and its surface is equal to at least 170 Pa mm${}^{-1}$. The samples were maintained at this pressure for one week, after which the mass of the samples remained constant. They were therefore considered to be dry.

### 2.3. Measuring Porosity and Gas Permeability

The porosity was measured following the testing method in [49]. It is expressed in percentage as follows:

(1) $$\phi =\frac{m_{3}-m_{1}}{m_{3}-m_{2}},$$

where $m_{1}$ is the oven-dried mass of the sample, $m_{2}$ is the mass of the sample in water, and $m_{3}$ is the mass in air of the water-saturated sample after vacuum.

On the other hand, the apparent permeability is a function of the pressure gradient and the gas flow. Assuming a uni-dimensional and quasi-static gas flow through a dry sample, Klinkenberg’s equation is written as follows:

(2) $$K_{\mathrm{app}}=K_{\mathrm{eff}}=K\left(1+\beta /P_{m}\right),$$

where $K_{\mathrm{app}}$ is apparent (or effective) gas permeability measured at the average gas pressure $P_{m}$ through the sample, β is Klinkenberg’s coefficient, and *K* is the intrinsic gas permeability. It is therefore possible to determine the permeability experimentally by circulating gas through a sample of known dimensions and measuring the associated pressure and flow rate. Dinitrogen gas has been flown through concrete samples. Different flow measurements at different pressure gradients are needed to determine multiple values of the apparent permeability. Finally, *K* can be obtained as the Y-intercept in a graph drawing the apparent gas permeability with respect to $\left(1/P_{m}\right)$.

### 2.4. Measuring Water Absorption

Capillary suction experiments were performed to study the impact of curing conditions and the proportion of GGBS on the porosity, intrinsic permeability, and water absorption of mortar samples. The method involves observing the saturation by capillary absorption of a dry sample in which the lower face is in contact with a wetting liquid (water). Water has a free surface with air; the pressure of water is then equal to that in the air, i.e., atmospheric pressure. The capillary imbibition tests were carried out on cylindrical samples, through the bottom face (vertical imbibition), while the top face remained open to the atmospheric pressure. The remaining surfaces of the samples were covered with plastic to let them impermeable and thus, the water could penetrate only through the bottom face. Due to capillary suction, water penetrates into the pores of the sample and progressively saturates it. The mass of samples was measured at different times and the evolution of the water absorption was recorded.

## 3. Modeling Approach

### 3.1. Mathematical Flow Model

The flow of water through concrete samples can be modeled using Darcy’s law. The volumetric water flux that penetrates a unit surface per unit time, *q*, is written as follows [46]:

(3) $$q=\frac{K\kappa }{\mu_{w}}\left(\nabla P_{L}-\rho_{w}gk\right),$$

where $P_{L}$, $\mu_{w}$, and $\rho_{w}$ represent pressure which will be later expressed in terms of water head *h*, dynamic viscosity, and density of liquid water, respectively. *g* denotes the acceleration due to gravity and *k* is the downward unit vector. *K* is the intrinsic permeability of the medium, which has area units and depends on the geometry of the porous media. κ (dimensionless) represents the relative permeability and relies on the water content θ of the porous media and can be expressed as a function of the capillary pressure. In this work, the Van Genuchten [50] formulation for concrete is adopted, i.e.,

(4) $$\begin{array}{ccc} \kappa & = & \theta_{e}^{\frac{1}{2}}{\left[1-{\left(1-\theta_{e}^{\frac{1}{m}}\right)}^{m}\right]}^{2}, \end{array}$$

(5) $$\begin{array}{ccc} \theta & = & \frac{\theta_{s}-\theta_{r}}{{\left|1+{\left(\alpha h\right)}^{n}\right|}^{m}}+\theta_{r}, \end{array}$$

where $\theta_{r}$ and $\theta_{s}$ are the residual and saturated water content, respectively. $\theta_{e}$ denotes the relative water content defined as:

(6) $$\theta_{e}=\frac{\theta -\theta_{r}}{\theta_{s}-\theta_{r}},$$

with *m*, *n*, and α are three fitting parameters. The usual assumption is considered in this work:

(7) $$m=1-\frac{1}{n}.$$

Finally, the time evolution of water content in a concrete sample is modeled using the following mass balance equation [46]:

(8) $$\frac{\partial \left(\rho_{w}\theta \right)}{\partial t}+\nabla \left(\rho_{w}q\right)=0,$$

where *t* denotes time. A finite element algorithm is developed and implemented to solve Equation (8). The model is capable to simulate various capillary absorption tests with reasonable computational cost.

### 3.2. Bayesian Inference

Bayesian inference is a statistical technique based on Bayes’ theorem to deal with inverse problems. The method has recently become very popular in many applications, such as geosciences, biosciences, and material sciences [51,52,53]. The first step in Bayesian inference is to choose the prior distribution for the unknown model parameters. The forward problem is then formulated using an appropriate likelihood function. Given observation data, the posterior distribution of the uncertain parameters is computed using Bayes’ rule [54]. In the following, we briefly go over this technique [55].

Let d be the vector of measured water content and Φ be the vector of model parameters. We consider the forward nonlinear model M represented by Equations (3)–(8) that forecasts the data as function of the model parameters such that:

(9) $$d\approx M\left(\Phi \right).$$

Applying Bayes’ rule gives:

(10) $$\Pi \left(\Phi |d\right)\propto L\left(d|\Phi \right)\;p\left(\Phi \right),$$

where $\Pi \left(\Phi |d\right)$ denotes the posterior, that represents the probability of occurrence of Φ given the data d. $L\left(d|\Phi \right)$ represents the likelihood function which is the probability of getting the data given the parameters vector Φ. Finally, $p\left(\Phi \right)$ is the prior of Φ, i.e., the *a priori* knowledge about the parameters.

To derive the likelihood function, we denote by ϵ=d−M the deviation between the observations and the model. The components of ϵ are assumed to be mutually independent and have the same probability distribution function with density $\rho_{\epsilon }$. Therefore, the likelihood function can be expressed as:

(11) $$L\left(d|\Phi \right)=\prod_{i}\rho_{\epsilon }\left[d_{i}-M_{i}\left(\Phi \right)\right].$$

Assuming that the errors $\epsilon_{i}$ follow a normal distribution with a mean of zero and variance of $\sigma^{2}$, i.e., $\epsilon_{i}∼N\left(0,\sigma^{2}\right)$, yields to the following likelihood function:

(12) $$L\left(d|\Phi \right)=\frac{1}{\sqrt{2\pi \sigma^{2}}}\prod_{i}\exp\left\{\frac{-{\left(d_{i}-M_{i}\left(\Phi \right)\right)}^{2}}{2\sigma^{2}}\right\}.$$

Using Bayes’ rule, the joint posterior can be written as:

(13) $$\Pi \left(\Phi |d\right)\propto \frac{1}{\sqrt{2\pi \sigma^{2}}}\prod_{i}\exp\left\{\frac{-{\left(d_{i}-M_{i}\left(\Phi \right)\right)}^{2}}{2\sigma^{2}}\right\}p\left(\Phi \right).$$

To compute the posterior, we still need to pick appropriate priors based on a priori knowledge about the parameters. In this study, a uniform prior for the parameters vector Φ is considered from the interval $\left[\Phi_{min}-\Phi_{max}\right]$, therefore:

(14) $$q\left(\Phi \right)=\left\{\begin{array}{cc} \frac{1}{\Phi_{max}-\Phi_{min}} & \mathrm{for}\;\Phi_{min}<\Phi <\Phi_{max},\\ 0 & \mathrm{otherwise}. \end{array}\right.$$

A popular computational strategy to sample the posterior is the MCMC method.

### 3.3. MCMC Sampling Procedure

In any Bayesian estimation procedure, we desire a search of the probability space for the most likely values of the retrieved state given a set of observations. However, it is not easy to analytically compute the posterior distribution. The MCMC method allows to directly sample the posterior following a Markov chain process and searches out areas of concentrated probability within the state space. In this paper, we use a version of MCMC based on the well-known Algorithm 1 [56,57] to accurately and efficiently sample the posterior distributions of the parameters.

In practice, the Markov chain starts from a set of parameters drawn from a bounded uniform probability distribution function, with bounds set to physically realistic values for each estimated parameter. In each MCMC iteration, a randomly-chosen parameter is generated using the prior distribution $p\left(\Phi \right)$. The model is then run using the new parameter values and new values of the state are generated and compared with the observations via a predetermined likelihood function. The new parameter is kept if the accepted probability is greater than a uniform random number, otherwise, the new value is rejected and the previous one is kept. The MCMC algorithm is run for many consecutive iterations to permit a comprehensive sampling of the state space, and a posterior probability density function is constructed for every parameter from the accepted values. The Metropolis-Hastings procedure is summarized in the following pseudo-code:
**Algorithm 1** Metropolis-Hastings algorithm.**Require:** 
Initial value $\Phi_{0}$ of the unknown parameters  1:Draw a new value $\Phi_{1}$ from the prior distribution *p*  2:Compute the joint posterior Π for both $\Phi_{0}$ and $\Phi_{1}$ using Equation (13)  3:Compute an acceptance probability: $\alpha =\frac{\Pi \left(\Phi_{1}|d\right)}{\Pi \left(\Phi_{0}|d\right)}$  4:Calculate a random number ε between 0 and 1  5:**if**α≥ε**then**  6:    Accept $\Phi_{1}$ and start step 1 with $\Phi_{1}$  7:**else**  8:    Start step 1 with $\Phi_{0}$  9:**end if**

## 4. Results and Discussion

### 4.1. Influence of Curing Conditions and GGBS Proportions on the Porosity

**Figure 1:**
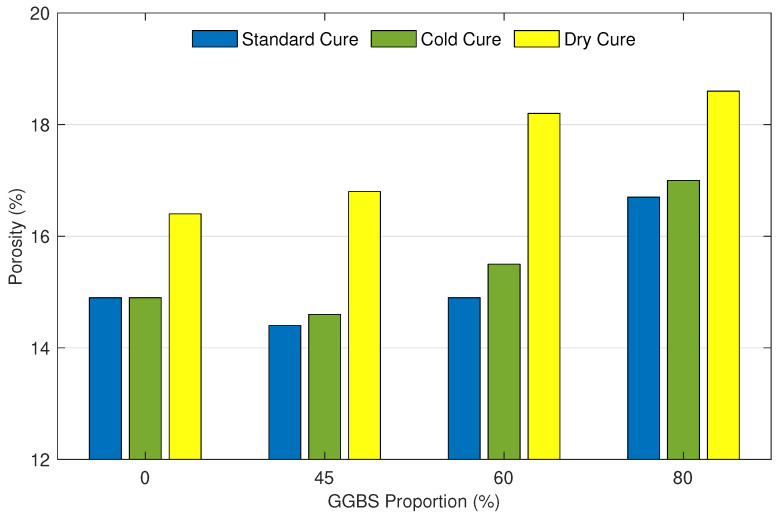
Porosity of mortar samples in function of their GGBS proportion and curing condition.

Figure 1 shows porosity values for the 12 samples measured by mercury intrusion porosimetry. The *X*-axis corresponds to the GGBS proportion to which each sample has been exposed. Based on the experimental results, the standard cure produced the lowest porosity with an average of 15.2%. The cold cure showed a slight change in the porosity compared to the standard cure. The average porosity is 15.5%, which is only 1.97% higher than the standard cure. However, the dry cure was found to have the highest porosity with an average of 18.1%, which is 19% higher than the standard cure.

Porosity values of mortar samples cured in standard condition with 0% (considered as the reference sample), 45%, 60%, and 80% of GGBS are 14.9, 14.4, 14.7, and 16.7%, respectively. It can be noticed that the lowest porosity, which is 3.36% lower than the reference sample, was observed for the sample with 45% of GGBS. The substitution of cement with 60% of GGBS showed a similar porosity value as the reference sample. With a further increase in GGBS replacement to 80%, porosity increased by 12%. A similar pattern was noticed for the cold cure with porosity values of 14.9, 14.7, 15.5, and 17%, respectively. Samples with 45% were close to the reference sample, beyond which the porosity started to increase. Samples incorporating 60% and 80% of GGBS surpassed the reference sample by 4% and 14%, respectively. Finally, the porosity values of samples cured under dry conditions are 17, 17.7, 18.3, and 19.3%, respectively. It is clear that the porosity increased with an increase in the GGBS amount. We observed that 0%, 45%, 60%, 80% of GGBS replacement resulted in 14.1%, 18.8%, 22.8%, and 29.5% increase in the porosity, respectively.

### 4.2. Influence of Curing Conditions and GGBS Proportions on the Permeability

**Figure 2:**
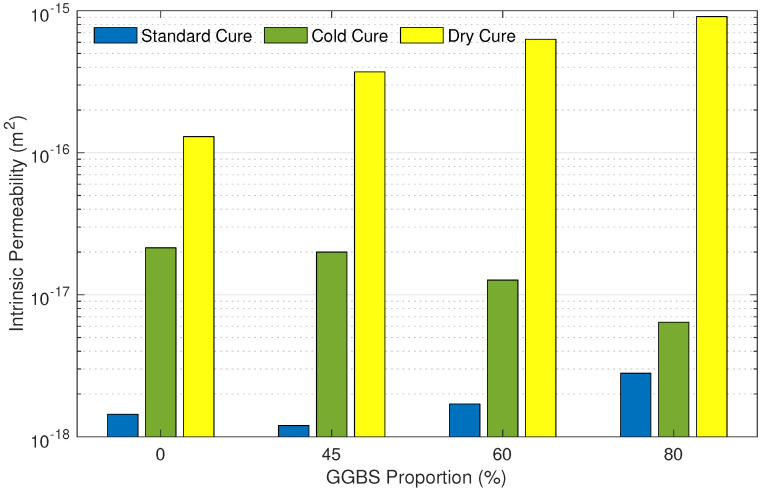
Intrinsic permeability of mortar samples in function of their GGBS proportion and curing condition.

Figure 2 displays intrinsic permeability values for the 12 samples. Intrinsic permeability values ranged between $1.2\times 10^{-18}$ and $9.1\times 10^{-16}$ m${}^{2}$. The standard cure had the lowest intrinsic permeability with an average of $1.8\times 10^{-18}$ m${}^{2}$. On the other hand, the dry cure developed the highest intrinsic permeability with an average of $5.1\times 10^{-16}$ m${}^{2}$, which is two orders of magnitude greater than the standard cure. Finally, the average intrinsic permeability of the cold cure was $1.5\times 10^{-17}$ m${}^{2}$, which is one order of magnitude greater than the standard cure.

Permeability values of samples cured in standard condition are $1.44\times 10^{-18}$, $1.2\times 10^{-18}$, $1.7\times 10^{-18}$, and $2.8\times 10^{-18}$ m${}^{2}$, for 0%, 45%, 60%, and 80% replacement of cement with GGBS, respectively. These results showed that the addition of 45% of GGBS gave the lowest permeability compared to the reference sample with an improvement of about 17%. However, permeability values were about 18% and 94% higher than the reference sample at 60% and 80% substitution of cement with GGBS, respectively. For the dry cure, permeability values were found to be $1.3\times 10^{-16}$, $3.71\times 10^{-16}$, $6.29\times 10^{-16}$, and $9.1\times 10^{-16}$ m${}^{2}$, respectively. Permeability values increased with an increase in the GGBS content. On the other hand, the observed trend is the opposite for samples cured under cold conditions. Permeability values are $2.14\times 10^{-17}$, $2\times 10^{-17}$, $1.27\times 10^{-17}$, and $6.4\times 10^{-18}$ m${}^{2}$, respectively. Unlike the standard and dry cures, results indicated that an increase in the replacement level of GGBS caused a decrease in permeability.

**Figure 3:**
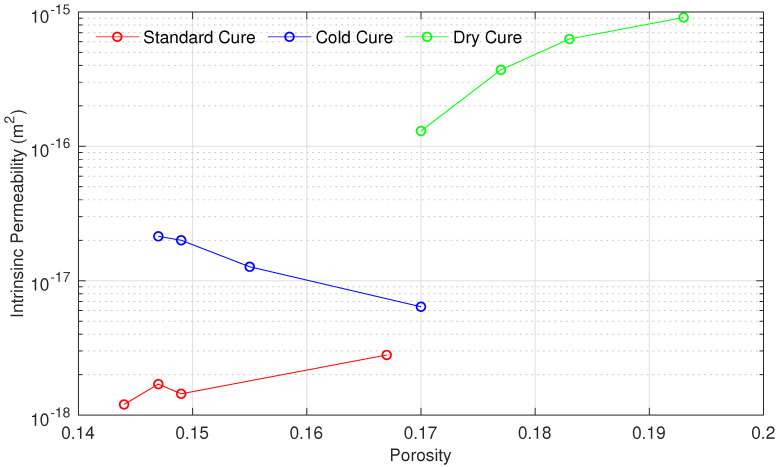
Intrinsic permeability of mortar samples in function of their porosity and curing condition.

Furthermore, the intrinsic permeability of all samples is plotted versus the porosity in Figure 3. According to these results, a clear correlation has been observed. In general, the permeability of samples cured in standard and dry conditions were higher for high porous samples. On the contrary, the permeability of the cold cure samples was lower for higher porous samples.

### 4.3. Influence of Curing Conditions and GGBS Proportions on the Water Absorption

**Figure 4:**
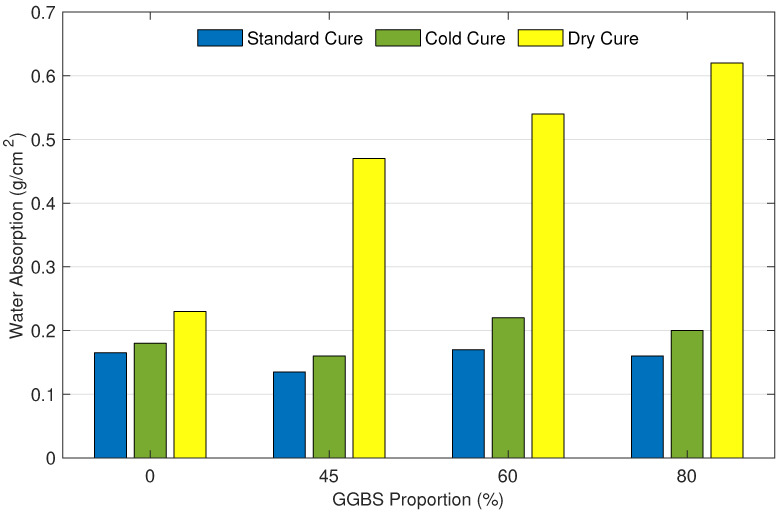
400 min water absorption by capillarity in mortar samples in function of their GGBS proportion and curing condition.

Water absorption values of mortar samples with different curing conditions and varying percentages of GGBS are presented in Figure 4. Samples cured in the standard condition presented the lowest water absorption, slightly lower than those cured in cold conditions. However, the high porosity and low relative humidity in the samples cured in dry conditions led to higher water absorption. Water absorption values in the samples cured in dry conditions are almost three times higher than those cured in standard conditions. Water absorption values for samples with 0%, 45%, 60%, and 80% of GGBS are 0.165, 0.135, 0.17, and 0.16 g·cm${}^{-2}$, respectively. These values for cold condition cured samples are 0.18, 0.16, 0.22, and 0.2 g·cm${}^{-2}$, respectively. Whereas, the water absorption for dry condition cured samples are 0.23, 0.47, 0.54, and 0.62 g·cm${}^{-2}$, respectively.

Figure 4 also shows that mortar samples containing 45% of GGBS performed better compared to those containing higher GGBS percentages. Among all samples, the maximum improvement in water absorption is caused by the standard cure sample with 45% of GGBS. The latest significantly improved the water absorption resistance by 18% compared to the reference sample. samples with 60% and 80% exhibited a similar water absorption resistance as the reference mix. Regarding the cold cure, samples with 45% of GGBS led to a 3% reduction in water absorption. Whereas the increase of GGBS to 60% and 80% showed a negative impact on the water absorption compared to the reference sample. Finally, the water absorption of all samples cured in dry conditions was higher in comparison with the reference sample. Results revealed that as the level of GGBS increases, so does the rate of water absorption.

### 4.4. Forward and Inverse Modeling

The numerical modeling approach seeks at imitating the experimental data collected during the capillary absorption test. The objective is to reproduce numerically the cumulative water absorption, which represents the water that crosses the mortar samples by capillary suction. With the aim of doing that, we implemented a one-dimensional finite element model that is able of simulating water absorption in mortar samples with a reasonable computational cost.

Assuming a nearly constant porosity and neglecting the impact of chemical reactions and according to the mathematical model represented by Equations (3)–(8), the flow of water that penetrates through the samples is a function of the parameters *K*, $\theta_{s}$, $\theta_{r}$, α, and *m*. The MCMC inverse procedure described above is then implemented to compute the optimal values of the parameters that best fit the capillary suction test data. As a way to simplify the parameter estimation process, we fix the value of $\theta_{s}$ and set it equal to the independently measured total porosity. We further impose $\theta_{r}$ to be equal to 0. To this end, the only remaining uncertain parameters in the MCMC inverse approach are *K*, α, and *m*.

### 4.5. Analysis of the MCMC Estimates

**Figure 5:**
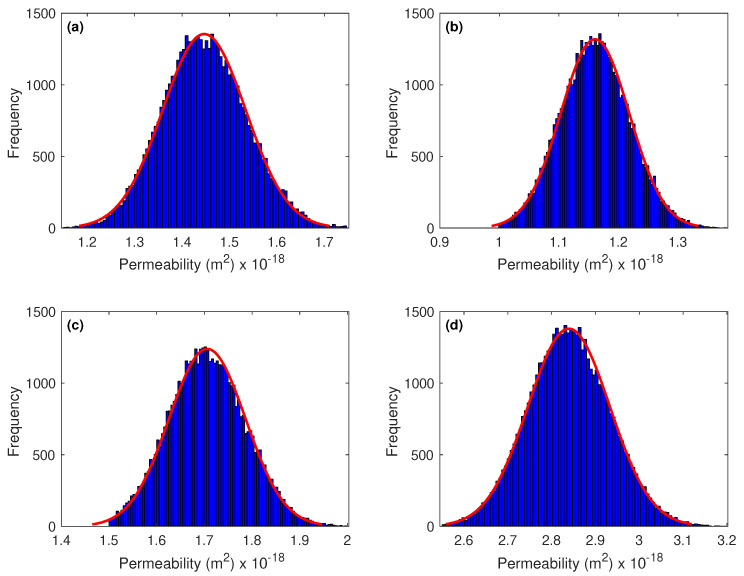
Posterior probability density functions of the intrinsic permeability resulting from the MCMC simulation. Estimates are shown for samples cured in standard condition. (**a**) GGBS0; (**b**) GGBS45; (**c**) GGBS60; and (**d**) GGBS80.

**Figure 6:**
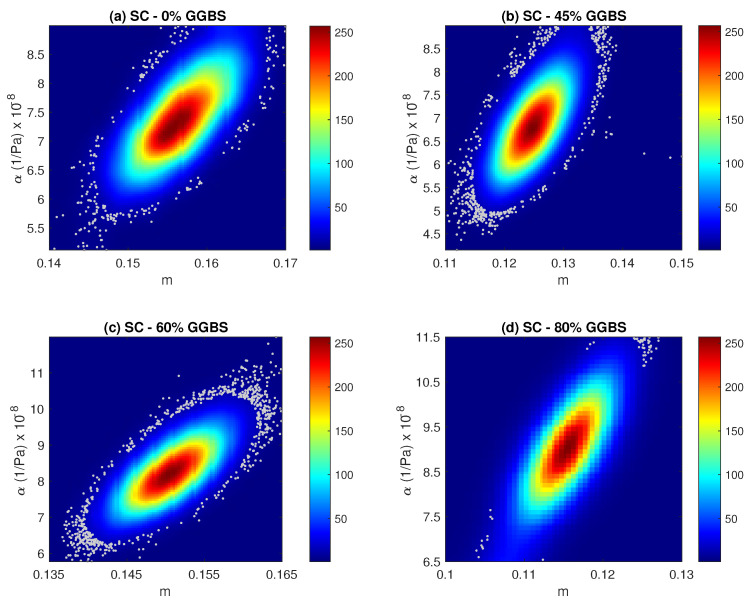
Two-dimensional joint posterior probability density functions of *m* and α resulting from the MCMC simulation. Estimates are shown for samples cured in standard condition.

Results of the Bayesian parameters inference for the standard cure are listed in Table 3 and displayed in Figure 5 and Figure 6 by means of histograms and probability density functions using kernel density estimation [58]. The two figures showed the most likely values for each parameters. Results are shown for samples cured in standard condition with 0%, 45%, 60%, and 80% of GGBS content. The posterior probability density functions of the four permeabilities (Figure 5) seem to follow Gaussian distributions with clear maximum *a posteriori* estimates of about $1.45\times 10^{-18}$ m${}^{2}$, $1.16\times 10^{-18}$ m${}^{2}$, $1.71\times 10^{-18}$ m${}^{2}$, and $2.84\times 10^{-18}$ m${}^{2}$. As can be seen, the results showed a notable consistency in the sense that the optimized values of the intrinsic permeability matched well those found in the experiments (see Table 3). Figure 6 depicted the two-dimensional joint probability density functions of the two parameters *m* and α. For the different samples, we observed posterior probability density functions that have well defined peaks of (m=0.156 and $\alpha =7.19\times 10^{-8}$ Pa${}^{-1}$), (m=0.125 and $\alpha =6.79\times 10^{-8}$ Pa${}^{-1}$), (m=0.15 and $\alpha =8.17\times 10^{-8}$ Pa${}^{-1}$), and (m=0.116 and $\alpha =9.02\times 10^{-8}$ Pa${}^{-1}$), respectively.

**Figure 7:**
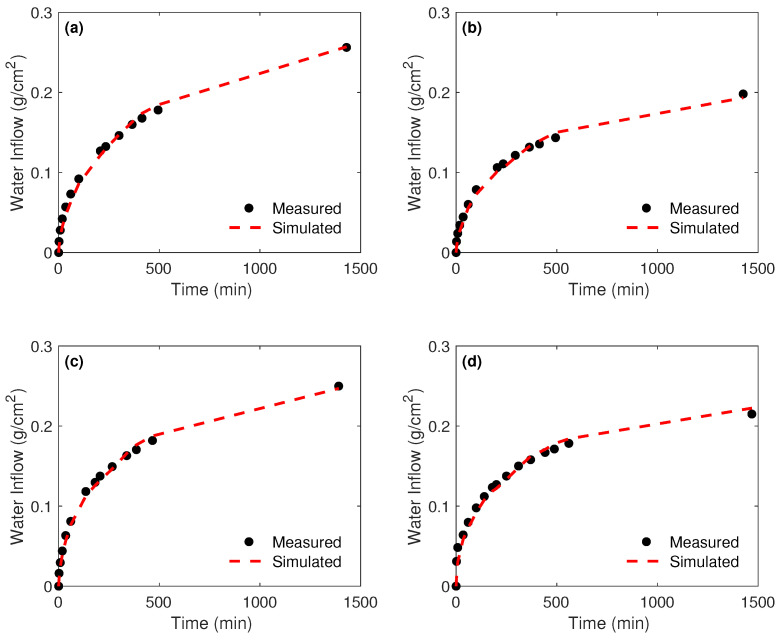
Cumulative measured and simulated water inflow. Results are shown for samples cured in standard condition. (**a**) GGBS0; (**b**) GGBS45; (**c**) GGBS60; and (**d**) GGBS80.

The cumulative measured and estimated water absorption of the four samples for a run time of about 24 h is plotted in Figure 7. The overall tendency of measured water absorption is nicely reproduced even though, as shown, the model tends to slightly underestimate the flux at the early stage of the experiment and to overestimate it after around 8 h. The RMSEs of the four samples shown in Table 3 are quite small and equal to 6.4, 5.3, 4.9, and $8.5\times 10^{-3}$, respectively. The accurate fitting of the water absorption in addition to the small values of the computed RMSEs reflects the reliability of the inverse technique and good recovery of the intrinsic permeability of the tested samples.

## 5. Conclusions

This paper highlights the impact of curing conditions on the durability performance of mortar samples incorporating selected GGBS proportions after a completely experimental and numerical study. A capillary suction experiment has been performed and a hydraulic model was implemented to accurately determine the hydraulic properties of the samples by inverse modeling using a Monte Carlo Markov Chain technique. Our main results are summarized as follows:

- Among the selected curing conditions, the performance of the standard curing condition is better than that of other curing conditions. Minimum porosity, intrinsic permeability, and water absorption were measured in samples cured in standard conditions, while maximum values were found in samples cured in dry conditions.
- Samples containing 45% of GGBS cured in standard condition slightly enhanced the porosity by 3% as compared to the reference sample. The porosity variation between the cold and standard cures was not that significant. On the other hand, the dry-cured samples didn’t show any improvement, whatever the replacement level of GGBS. Porosity values increased with an increase in GGBS content.
- The permeability was improved by 17% with the inclusion of 45% of GGBS in the standard curing samples. All other samples presented higher permeability values compared to the reference sample. Cold curing samples showed a significant decrease in permeability values with an increase in GGBS replacement, while the opposite tendency was observed for dry curing samples.
- In terms of water absorption, maximum improvement of 18% was found in samples cured in standard condition with 45% of GGBS. With a further increase in GGBS replacement, standard curing samples exhibited almost a similar water absorption to that of the reference sample. For cold-curing samples with 45% of GGBS, we also observed a little improvement of 3%. Beyond 45%, a slight drop in water absorption resistance was noticed. Ultimately, dry curing samples revealed a significant increase in water absorption with an increase in GGBS content.
- Numerical inverse modeling showed a good agreement between measured and estimated intrinsic permeability. Moreover, the simulated cumulative weights of the samples over time fitted well with those measured. The corresponding average RMSE is around $6\times 10^{-3}$. Such promising results demonstrate the capability of the proposed inference approach to accurately predict the hydraulic parameters of the samples from extremely short capillary suction experiments and simulate water flow through mortar samples.

## Figures and Tables

**Table 1:** Chemical composition of cement and GGBS (%).

Material	SiO${}_{2}$	Al${}_{2}$O${}_{3}$	Fe${}_{2}$O${}_{3}$	CaO	Na${}_{2}$O	K${}_{2}$O	MgO	SO${}_{3}$
CEM I 52.5 N	20.1	4.8	3.4	63.6	0.1	1.0	1.3	3.1
GGBS	36.2	11.5	0.3	41.3	0.1	0.4	7.3	3.7

**Table 2:** Mortar mixes and corresponding content proportions.

Mix	Cement (kg/m${}^{3}$)	GGBS (kg/m${}^{3}$)	Water (kg/m${}^{3}$)	Sand (kg/m${}^{3}$)	w/c
CEM-GGBS 0%	450	0	202.5	1350	0.45
CEM-GGBS 45%	247.5	202.5	202.5	1350	0.45
CEM-GGBS 60%	180	270	202.5	1350	0.45
CEM-GGBS 80%	90	360	202.5	1350	0.45

**Table 3:** Parameter values estimated using MCMC and the associated water inflow RMSEs of mortar samples subjected to standard curing condition.

Sample	$K\times 10^{-18}\;($m${}^{2})$	$K\times 10^{-18}\;($m${}^{2})$	$\alpha \times 10^{-8}\;($Pa${}^{-1})$	*m*	$\mathrm{RMSE}\times 10^{-3}$
	Experimental	Numerical			
CEM-GGBS 0%	1.44	1.45	7.19	0.156	6.4
CEM-GGBS 45%	1.20	1.16	6.79	0.125	5.3
CEM-GGBS 60%	1.70	1.71	8.17	0.150	4.9
CEM-GGBS 80%	2.80	2.84	9.02	0.116	8.5

## Data Availability

Not applicable.
